# Gender, health and the 2030 agenda for sustainable development

**DOI:** 10.2471/BLT.18.211607

**Published:** 2018-06-12

**Authors:** Mary Manandhar, Sarah Hawkes, Kent Buse, Elias Nosrati, Veronica Magar

**Affiliations:** a Gender, Equity and Human Rights Team, World Health Organization, avenue Appia 20, 1211 Geneva 27, Switzerland.; b Centre for Gender and Global Health, Institute for Global Health, Faculty of Population Health Sciences, University College London, London, England.; c Strategic Policy Directions, Joint United Nations Programme on HIV/AIDS, Geneva, Switzerland.; d Department of Sociology, University of Cambridge, Cambridge, England.

## Abstract

Gender refers to the social relationships between males and females in terms of their roles, behaviours, activities, attributes and opportunities, and which are based on different levels of power. Gender interacts with, but is distinct from, the binary categories of biological sex. In this paper we consider how gender interacts with the 2030 agenda for sustainable development, including sustainable development goal (SDG) 3 and its targets for health and well-being, and the impact on health equity. We propose a conceptual framework for understanding the interactions between gender (SDG 5) and health (SDG 3) and 13 other SDGs, which influence health outcomes. We explore the empirical evidence for these interactions in relation to three domains of gender and health: gender as a social determinant of health; gender as a driver of health behaviours; and the gendered response of health systems. The paper highlights the complex relationship between health and gender, and how these domains interact with the broad 2030 agenda. Across all three domains (social determinants, health behaviours and health system), we find evidence of the links between gender, health and other SDGs. For example, education (SDG 4) has a measurable impact on health outcomes of women and children, while decent work (SDG 8) affects the rates of occupation-related morbidity and mortality, for both men and women. We propose concerted and collaborative actions across the interlinked SDGs to deliver health equity, health and well-being for all, as well as to enhance gender equality and women’s empowerment. These proposals are summarized in an agenda for action.

## Résumé

Le genre fait référence aux relations sociales entre les hommes et les femmes pour ce qui est de leurs rôles, comportements, activités, attributs et opportunités, qui reposent sur différents niveaux de pouvoir. Le genre interagit avec les catégories binaires du sexe biologique mais diffère de celles-ci. Dans cet article, nous nous intéressons aux interactions entre le genre et le Programme de développement durable à l'horizon 2030, notamment l'objectif de développement durable (ODD) 3 et ses cibles en matière de santé et de bien-être, ainsi qu'à son impact sur l'équité dans le domaine de la santé. Nous proposons un cadre conceptuel pour comprendre les interactions entre le genre (ODD 5) et la santé (ODD 3) ainsi que 13 autres ODD qui influencent la santé. Nous examinons les données empiriques afin de relever ces interactions dans trois domaines du genre et de la santé: le genre comme déterminant social de la santé; le genre comme facteur de comportements liés à la santé; et la réponse sexospécifique des systèmes de santé. Cet article souligne la relation complexe entre la santé et le genre, et la manière dont ces trois domaines interagissent avec le Programme 2030 dans son ensemble. Dans ces trois domaines (déterminants sociaux, comportements liés à la santé et systèmes de santé), les données révèlent les liens entre le genre, la santé et d'autres ODD. L'éducation (ODD 4), par exemple, a un impact mesurable sur la santé des femmes et des enfants, tandis qu'un travail décent (ODD 8) affecte le taux de morbidité et de mortalité pour cause professionnelle, aussi bien chez les hommes que chez les femmes. Nous proposons des actions collaboratives et concertées vis-à-vis de ces ODD interdépendants afin d'assurer l'équité en matière de santé ainsi que la santé et le bien-être pour tous, et de renforcer l'égalité des genres et l'autonomisation des femmes. Ces propositions sont résumées dans un programme d'action.

## Resumen

El género hace referencia a las relaciones sociales entre hombres y mujeres en términos de roles, comportamientos, actividades, atributos y oportunidades, y se basan en diferentes niveles de poder. El género interactúa con, pero es distinto de, las categorías binarias del sexo biológico. En este documento, consideramos cómo el género interactúa con la agenda 2030 para el desarrollo sostenible, incluidos los Objetivos de Desarrollo Sostenible (ODS) 3 y sus objetivos para la salud y el bienestar, y el impacto en la equidad en salud. Proponemos un marco conceptual para comprender las interacciones entre género (ODS 5) y salud (ODS 3) y otros 13 ODS, que influyen en los resultados de salud. Exploramos la evidencia empírica de estas interacciones en relación con tres dominios de género y salud: el género como determinante social de la salud; el género como conductor de conductas de salud; y la respuesta de género de los sistemas de salud. El documento destaca la compleja relación entre salud y género, y cómo estos dominios interactúan con la amplia agenda de 2030. A través de los tres dominios (determinantes sociales, comportamientos de salud y sistema de salud), encontramos evidencia de los vínculos entre género, salud y otros ODS. Por ejemplo, la educación (ODS 4) tiene un impacto cuantificable en los resultados de salud de mujeres y niños, mientras que el trabajo decente (ODS 8) afecta las tasas de morbilidad y mortalidad relacionadas con la ocupación, tanto para hombres como para mujeres. Proponemos acciones coordinadas y colaborativas entre los ODS interconectados para generar equidad en salud, salud y bienestar para todos, así como para mejorar la igualdad de género y el empoderamiento de las mujeres. Estas propuestas se resumen en una agenda de acción.

## ملخص

يشير نوع الجنس إلى العلاقات الاجتماعية بين الذكور والإناث من حيث أدوارهم وسلوكياتهم وأنشطتهم وما يتمتعون به من صفات وما يحظون به من فرص، وهو ما يستند إلى مستويات مختلفة من السلطة. ****يتفاعل نوع الجنس مع فئات ثنائية من الجنس البيولوجي، ولكنه يأخذ شكلاً مختلفاً. ****سوف نناقش في هذه الورقة البحثية كيفية تفاعل نوع الجنس مع جدول أعمال التنمية المستدامة لعام 2030، بما في ذلك الهدف 3 للتنمية المستدامة (SDG)، وغاياته المتعلقة بالصحة والرفاهية، وكذلك التأثير على العدالة الصحية. ****نحن نقترح إطارًا مفاهيميًا لفهم التفاعلات بين نوع الجنس (الهدف 5 من أهداف التنمية المستدامة) والصحة (الهدف 3 من أهداف التنمية المستدامة) و13 هدفاً آخراً للتنمية المستدامة تؤثر على النتائج الصحية. ****سوف نستكشف الدليل التجريبي لهذه التفاعلات فيما يتعلق بثلاثة مجالات لنوع الجنس والصحة: ****نوع **​ **الجنس كمحدد اجتماعي للصحة، ونوع الجنس كمحرك للسلوكيات الصحية؛ واستجابة نوع الجنس للنظم الصحية. ****تسلط الورقة البحثية الضوء على العلاقة المعقدة بين الصحة ونوع الجنس، وكيف تتفاعل هذه المجالات مع جدول الأعمال الموسّع لعام 2030. ****في جميع هذه المجالات الثلاثة (المحددات الاجتماعية والسلوكيات الصحية والنظام الصحي)، نجد أدلة على الروابط بين نوع الجنس والصحة والأهداف الأخرى للتنمية المستدامة. ****فمثلاً، يؤثر التعليم (الهدف 4 من أهداف التنمية المستدامة) على النتائج الصحية للنساء والأطفال بشكل يمكن قياسه، بينما يؤثر العمل اللائق (الهدف 8 من أهداف التنمية المستدامة) على معدلات الإصابة بالأمراض والوفيات المرتبطة بالوظيفة، لكل من الرجال والنساء. ****ونحن نقترح اتخاذ إجراءات تعاونية ومتفق عليها في إطار أهداف التنمية المستدامة المترابطة فيما بينها، وذلك لتحقيق المساواة الصحية، والصحة والرفاهية للجميع، فضلاً عن تعزيز المساواة بين الجنسين وتمكين المرأة. ****يتم تلخيص هذه المقترحات في جدول أعمال لاتخاذ الإجراءات اللازمة.

## 摘要

性别是指基于社会分工、行为、活动、属性和机会方面的不同水平，从而形成的男女之间的社会关系。性别与二元分类的生理性别相互作用，但是二者并不相同。在本文中，我们考虑性别如何与 2030 年可持续发展议程（包括可持续发展目标 3） 及其良好健康与福祉的目标相互作用，以及对医疗平等的影响。我们提出一个概念框架，用以理解性别（可持续发展目标 5）和健康（可持续发展目标 3）以及影响健康结果的其他 13 个可持续发展目标之间的相互作用。我们探究关于性别和健康的三个领域（作为社会健康决定因素的性别、作为健康行为驱动因素的性别和卫生系统对不同性别的回应）相互作用的经验证据。本文强调健康和性别之间的复杂关系，以及这些领域如何与广泛意义上的 2030 年可持续发展议程相互作用。纵观这三个领域（社会决定因素、健康行为和卫生系统），我们发现了性别、健康和其他可持续发展目标之间存在联系的证据。例如，优质教育（可持续发展目标 4）对女性和儿童的健康结果有可估量的影响，而体面工作（可持续发展目标 8）会影响男性和女性因工作而导致的发病率和死亡率。我们提出为实现环环相扣的可持续发展目标采取协调一致和协作行动，以实现医疗平等，让所有人享有良好健康与福祉，加强性别平等并为女性赋权。这些议案会归纳入行动议程中。

## Резюме

Гендер относится к социальному взаимодействию между мужчинами и женщинами с точки зрения их ролей, поведения, деятельности, характеристик и возможностей, которое основано на разных уровнях власти. Гендер взаимосвязан с бинарными категориями биологического пола, но отличается от него. В этом документе авторы рассматривают вопрос о том, как гендерные аспекты связаны с повесткой дня в области устойчивого развития на период до 2030 года, включая 3-ю цель в области устойчивого развития (ЦУР) и ее задачи в области здоровья и благополучия, а также влияние на обеспечение равенства в вопросах здравоохранения. Авторы предлагают концептуальную основу для понимания взаимосвязи между гендерными аспектами (ЦУР 5), здоровьем (ЦУР 3) и 13 другими ЦУР, которые влияют на результаты в сфере здравоохранения. Авторы изучают эмпирические данные для этих взаимосвязей в отношении трех областей гендера и здоровья: гендерный фактор как социальная детерминанта здоровья, гендерный фактор как движущая сила поведения в отношении здоровья и то, как гендер определяет реакцию систем здравоохранения. В документе подчеркивается сложная взаимосвязь между здоровьем и гендером и то, как эти области взаимосвязаны с обширной повесткой дня на период до 2030 года. Во всех трех областях (социальные детерминанты, поведение в отношении здоровья и система здравоохранения) авторы выявили доказательства взаимосвязи между гендером, здоровьем и другими ЦУР. Например, образование (ЦУР 4) оказывает ощутимое влияние на результаты в отношении здоровья женщин и детей, в то время как наличие достойной работы (ЦУР 8) влияет на показатели заболеваемости и смертности, связанные с профессиональной занятостью, как для мужчин, так и для женщин. Авторы предлагают согласованные и совместные действия в рамках взаимосвязанных ЦУР для обеспечения равенства в вопросах здравоохранения, здоровья и благополучия для всех, а также для укрепления гендерного равенства и расширения прав и возможностей женщин. Эти предложения изложены в плане действий.

## Introduction

Globally, the average life expectancy gap between men and women is 4.6 years, with women outliving men in all countries, and a gap of over 10 years in some cases.^1^ In addition, the global burden of disease disproportionately affects men in terms of disability-adjusted life years,^2^ although women are more likely to spend a longer time living with a disability.^3^

In part, these differences may be due to the impact of sex: biological differences between males and females in growth, metabolism, reproductive cycles, sex hormones and ageing processes.^4^ Even when men and women are equally exposed to a risk or disease, the health consequences may be different for each sex. For example, among men and women who smoke tobacco, women appear to develop severe chronic obstructive pulmonary disease at younger ages than men and with lower cumulative cigarette smoke exposure.^5^ However, biological explanations for differences between men and women have limited powers to explain the worldwide differences in health outcomes throughout human history, including in times of rapid demographic and epidemiological transition.^6^ These differences are largely due to the social phenomenon of gender.^7^

Gender refers to the roles, behaviours, activities, attributes and opportunities that any society considers appropriate for boys and girls, and men and women. Gender also refers to the relationships between people and can reflect the distribution of power within those relationships. An understanding of gender requires understanding the complex social processes through which people are defined and linked and how this evolves over time. These processes operate at an interpersonal level, at an institutional level and across wider society, in government, the institutions of the state and whole economies. At all these levels, gender is an important, but modifiable determinant of health across the life course.^8^ Taking this perspective avoids polarization between men (as perpetrator) and women (as victim), and recognizes and addresses the needs of transgender people.^9^

Gender intersects with other drivers of inequities, discrimination, marginalization and social exclusion, which have complex effects on health and well-being. These intersectional drivers include ethnicity, class, socioeconomic status, disability, age, geographical location, sexual orientation and sexual identity. Intersectionality refers to the meaning and relationship between these factors, in processes and systems of power at the individual, institutional and global levels.^10^ The concept of intersectionality builds on, and extends, a gendered analysis of health, by identifying how relationships of power interact with these drivers and gender at different levels.^11^

This paper reflects on the relationship between gender and health in the context of the sustainable development goals (SDGs). The design of the goals is based on interdependence – meaning that no single goal can be achieved without action in other goals. We consider SDG 5 (that is, achieve gender equality and empower all women and girls) as it interacts with SDG 3 (that is, ensure healthy lives and promote well-being for all at all ages), and how both gender and health intersect across multiple other SDGs in ways that can either hinder or enhance health equity. 

## Gender and health

Beyond SDGs 3 and 5, gender equality is a cross-cutting feature of *Transforming our world: the 2030 agenda for sustainable development*^12^ and is key to realizing women’s and girls’ rights and catalysing progress across all SDGs. There are six gender-specific indicators within SDG 3 on health: (i) maternal mortality ratio; (ii) births attended by skilled health personnel; (iii) new human immunodeficiency virus (HIV) infections, by sex; (iv) satisfactory family planning with modern methods; (v) adolescent birth rate; and (vi) coverage of essential health services, including reproductive and maternal health. Aside from the SDG 3 targets, SDG 5, which includes the elimination of violence against women and girls, has important implications for health.

Other SDGs have been categorized by the United Nations Entity for Gender Equality and the Empowerment of Women (UN Women) as either gender-sensitive or gender-sparse,^11^ reflecting the importance of viewing the entire SDG framework through a gender lens. We apply this wider gender perspective to improve understanding and to inform action on health, focusing on the impact on health outcomes.

Conceptually, gender has been described as influencing health and well-being across three domains: (i) through its interaction with the social, economic and commercial determinants of health; (ii) via health behaviours that are protective of, or detrimental to, health outcomes; and (iii) in terms of how the health system responds to gender, including how it affects the financing of and access to quality health care.^13^^,^^14^

**Fig. 1:**
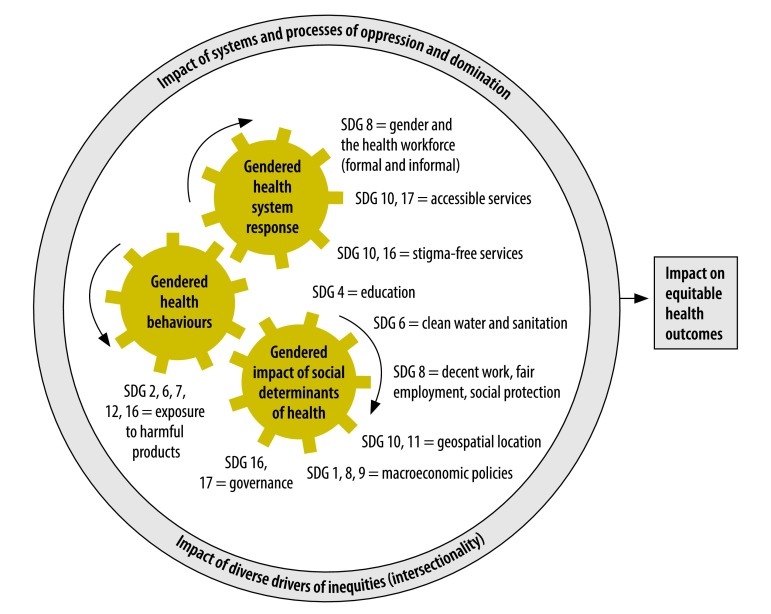
Conceptual framework to show interactions between sustainable development goals 3 (health) and 5 (gender) with other global goals across three domains of gender and health

We have extended this framework to outline how several different SDGs impact on each of these three domains (Fig. 1). The domains interact with each other; for example, governance (social determinants domain) determines responses by the health system to a situation (health system domain). The domains also all operate in a wider sociopolitical, cultural and historical context, shaping a range of intersectional drivers of inequalities, exclusion and discrimination that operate alongside gender. Using this framework, we provide evidence of the linkages across gender and the health-related targets of a range of SDGs including, but not limited to, the SDGs on health and gender (Table 1).

SDG: sustainable development goal.

**Table 1:** Selected examples of interactions of sustainable development goals 3 (health) and 5 (gender) with other global goals across three domains of gender and health

Health domains (SDG 3)	**Links to gender (SDG 5)**	Selected examples	Links to other SDGs	Sources^a^
**Social determinants domain**				
Premature mortality, preventable morbidity	Gendered differences in income are often associated with poorer health outcomes	Globally there are 122 women aged 25–34 years living in extreme poverty for every 100 men of the same age group. Up to 30% of income inequality is due to inequality within households, including between men and women, with women more likely than men to live on below 50% of the median national income. Women accrue fewer years of formal paid employment than men, due to childbirth and their gendered role as main carers. Wage depression and gender pay-gaps are widespread, leaving women less well-off than men. In India, Dalit women, who are lower-caste women in a poorer socioeconomic population group, have a life expectancy 14.6 years shorter than upper-caste women. In both stable and crisis settings, older age, female sex, economic deprivation and rural residency are frequently associated with poor health outcomes	1 (no poverty); 7 (affordable & clean energy); 8 (decent work and economic growth); 10 (reduced inequalities); 13 (climate action)	Institute for Health Metrics and Evaluation, 2018^2^; UN Women, 2018; Overseas Development Institute, 2014^15^
	Gendered differences in occupation can lead to different exposure to risks of premature mortality and preventable morbidity	Health risks are higher for men working in extractive and construction industries and road transport jobs or drafted into armed conflicts. Risks of indoor air pollution are higher for women working in the home due to the use of unclean combustible fuels caused an estimated 4.3 million deaths in 2012 and women and girls accounted for 6 out of every 10 of these deaths. Women working in flower farming and their children and newborns have greater exposure to pesticides and chemical residues than men do		
	Climate change can have a disproportionate impact on women	Girls and women in climate-related disasters such as flooding are at greater risk than boys and men because they are less likely to be able to swim		
Mental and physical health	Girl’s education and their lower socioeconomic position can impact on health outcomes	Worldwide, 15 million girls of primary school age will never get the chance to learn to read or write in primary school compared with 10 million boys. The proportion of time spent on unpaid domestic and care work by women is 2.6 times greater than for men	1 (no poverty); 4 (quality education); 6 (clean water and sanitation); 8 (decent work and economic growth); 16 (peace; justice and strong institutions)	UN Women, 2018^11^
Nutrition	Gendered norms and practices about food distribution often disadvantage girls and women	Women are up to 11 percentage points more likely than men to report food insecurity (i.e. secure access to sufficient amounts of safe and nutritious food for normal growth and development and an active and healthy life). Within households men and boys are often given greater quantities of food and more nutritious food than women and girls	2 (zero hunger); 6 (clean water and sanitation)	UN Women, 2018^11^
Communicable diseases	Gendered patterns in exposure can make women more vulnerable to communicable diseases	Schoolteachers, who are more likely to be female, are at higher risk for influenza due to greater contact with children; men are more susceptible to H5N1 influenza through work in fowl and poultry slaughter and processing industries. Women may face increased exposure to infection with malaria linked to gender-defined occupations (e.g. seeking water and fuel or agricultural tasks, at peak biting times of mosquitos). Men, however, may have higher malaria risk due to working in fields, mines, ponds and other high-exposure locations. In the 2013–2016 Ebola virus disease epidemic, women were found to be at higher risk of infection due to caring roles within the household, whereas men were more at risk due to their involvement in burial rituals	8 (decent work and economic growth)	WHO, 2010^16^; WHO, 2007^17^; Davies and Bennett, 2016^18^
**Health behaviours domain**				
Noncommunicable diseases	Gendered patterns of risk exposure to unhealthy products (especially alcohol and tobacco) tend to increase morbidity and mortality in men	Gendered patterns in exposure to noncommunicable disease risk and poor health-care-seeking behaviours contributes to excess male mortality Corporate strategies towards expanding markets for smoking and alcohol use are increasingly designed to include women, particularly younger women, from low- and middle- income countries	2 (zero hunger); 12 (responsible consumption and production)	UN Women, 2018^11^
Communicable diseases	Gender norms can affect the uptake of preventive services by women	Women are less likely to accept services from male community drug distributors for neglected tropical diseases when male members of the household are not present	8 (decent work and economic growth)	Theobald et al. 2017^19^
**Health system domain**				
Universal health coverage	Gendered patterns of employment may affect women’s access to health-care services	Women have longer lifespans, but often fewer years of paid employment and accrued pension than men. Therefore women may have less access to health-care services in their old age as a result of smaller contributory pensions	1 (no poverty); 4 (quality education); 8 (decent work and economic growth); 10 (reduced inequalities); 16 (peace, justice and strong institutions); 17 (partnerships for the goals)	UN Women, 2018^11^; Theobald et al., 2017^19^; Davies & Bennett, 2016^18^; Raine, 2000^20^; Dey et al., 2009^21^; Regitz-Zagrosek, 2011^22^
	Gendered power dynamics within the household can determine spending on health care	A lower percentage of women than men have control over how they spend their own earnings. Women have lower purchasing power than men when men act as the head of the household or when there is no male earner in the household. This affects women’s risk of experiencing catastrophic health spending		
	Gendered stereotyping by health-care providers and gendered differences in presentation of diseases can affect diagnostic and treatment pathways	Heart disease is often construed as a disease typically affecting men. Men are more often referred to specialists than women for certain conditions (cardiac arrhythmias, cerebrovascular disease, vascular surgery, hip replacement and heart transplantation). Heart disease also presents differently by sex. The result is both mis- and under-diagnosis in women, which may result in more adverse outcomes in women with cardiovascular symptoms		
	Gender norms can affect the uptake of services by women	Deploying men as community distributors of drugs for neglected tropical diseases can negatively affect coverage of services for women		
	Gendered patterns of work can affect access to public health interventions	In adult vaccinations programmes, men who are working outside the district in a non-endemic area can miss out on programme interventions		
	Health systems may not take account of how unequal gender norms, roles and relations affect health	Analysis of the 2013–2016 Ebola virus disease epidemic revealed a lack of data indicators disaggregated by sex. The early response to the epidemic ignored the different roles of men and women (e.g. men buried the dead, while women cared for sick people at home) and hence the different potential pathways for transmission of the virus		
Health workforce	Gender affects the health workforce itself	Women who have to work late or are away from home for long periods of time can suffer psychological and physical abuse from husbands or mothers-in-law for taking time away from household caretaking roles	8 (decent work and economic growth)	UN, 2017^23^ WHO, 2015^24^; WHO-Europe, 2015^25^; WHO, 2017^26^
	Discrimination in health-care settings can lead to gaps in coverage	Gender discrimination interacts with multiple types of discrimination based on other drivers of inequities. Older people can face discrimination accessing health services. Ethnic minorities, such as Roma, and migrants (especially illegal migrants) and patients with long-term illness faced discriminatory barriers accessing health services in Greece		
	Gendered institutional responses can affect people’s physical and mental health	Gender bias and the wider stigma and discrimination in society deters transgender populations and men with human immunodeficiency virus from seeking care		
Governance	Lack of gender parity in decision-making positions and leadership in the health workforce can affect women’s access to health	There is a lack of women in senior management positions in district or village level clinics and low participation of women in subnational health committees. Decentralized health services, particularly in areas where health and other decisions are predominantly made by men, can hinder choices, information and service access for women and girls, especially sexual and reproductive services. Health-system responses during the 2016 Zika virus emergency response did not take account of unequal gender norms, roles and relations. Women who were already marginalized were advised to avoid pregnancy, apparently without an acknowledgement of their difficulties in access to contraceptives, sex education and safe abortion practices	8 (decent work and economic growth); 16 (peace, justice and strong institutions)	Scott et al., 2017^27^; Global Health 50/50, 2018^28^; Davies & Bennett, 2016^18^; Langer, 2015^26^

SDG: sustainable development goal; UN: United Nations; WHO: World Health Organization; WHO-Europe: World Health Organization Regional Office for Europe.

^a^ Some of the examples used do not refer to specific research studies but to a combination of research and analysis by organizations such as UN Women. It is outside the scope of this article to cite to every source of evidence. Readers should consult the reference list of cited reports for more information.

## Social determinants

The landmark 2008 Commission on the Social Determinants of Health^29^ reported that the global burden of disease, and major causes of health inequities, arise from the different conditions in which people are born, grow, live, work and age. These conditions are affected by inequities in power, money and resources, and all of them are affected by gender. The underlying contexts of socioeconomics and politics (governance, macroeconomic policies, cultural norms and social values), social position (education, occupation, ethnicity and gender) and wider social environment (community cohesion, and social group and individual behaviours) are all represented as targets of SDG action.

For example, girls’ access to education (linked to SDG 4, quality education) has a measurable impact on their own, and their children’s, health outcomes.^30^ The beneficial effect of education can be affected by macro-level political and economic forces which result in contraction of welfare provision (including health-system cuts), wage depression and food insecurity. These factors are included in SDGs 1 (no poverty); 2 (zero hunger), 8 (decent work and economic growth) and 10 (reduced inequalities) and all have been shown to have adverse effects on child and maternal health.^31^ Similarly, rapid privatization programmes in post-communist countries (linked to SDG 9, industry, renovation and infrastructure) have shaped gendered differences in mortality over and above the micro-level determinants of health.^32^ SDG 8 (for promoting economic growth, full and productive employment and decent work for all) is strongly linked to health and gender.^33^^,^^34^ Examples of interactions among SDGs 3 and 5 and other SDGs are provided in Table 1.

## Health behaviours

Sociocultural norms and related patterns of behaviours differ according to gender. These can affect health behaviours in different ways for a variety of conditions. There is growing recognition of the roles in risk-taking played by sociocultural norms and related qualities and patterns of behaviours traditionally associated with being a man (referred to as masculinity).^35^ The behaviours include avoiding condom use,^36^ greater use of harmful substances^37^ and lower rates of seeking testing and treatment for HIV. These expressions of masculinity can also impact on the health of girls and women, for example through violence, sexually transmitted infections and unwanted pregnancies.^36^ In terms of differential exposure to products that are harmful to health (SDG 12, responsible consumption and production), longitudinal studies point to tobacco smoking and alcohol consumption as key contributors to excess male mortality.^38^ However, current patterns in the consumption of tobacco and alcohol by sex are changing, with commercial strategies to expand sales of products increasingly targeting women, particularly in low- and middle-income countries.^39^^,^^40^ There is evidence that the proportion of females using alcohol is increasing, notably among young adults, with increases in related harm.^41^

Aside from differences in health behaviours related to exposure to a range of illnesses, gender is also associated with responses to symptoms and signs of illness. Studies have shown that women are more likely to seek health care than men do, even after adjusting for reproductive health consultations.^42^ Other factors, however, such as women’s lack of autonomy to make out-of-pocket payments for health care,^43^ can inhibit women’s access to health care.

## Health systems

Health systems themselves are not gender-neutral.^44^ The role of gender within health systems relates to concepts of universal health coverage (SDG 3), pathways of care including the impact of gender stereotypes and gender-related stigma that drive inequalities (SDG 10, reduced inequalities), principles of accountability and inclusivity (SDG 16, peace, justice and strong institutions), and the gendered experience of the health workforce itself (SDG 8, decent work and economic growth; SDG 16, peace, justice and strong institutions). However, there are concerns that decision-makers in the global health system are not well-prepared to understand,^45^ and effectively respond to, the structural, social, commercial and frequently gendered determinants of the major emerging burdens of disease. This is especially true for those determinants associated with environmental degradation, poor urban planning and unsustainable patterns of consumption. A recent analysis revealed the extent and nature of low gender awareness in the emergency responses to Ebola and Zika virus epidemics and in longer-term planning for health-system resilience (Table 1).^18^

It has been estimated that at least half of the world’s 7.3 billion people do not receive the essential health services they need, with substantial unmet need for a range of specific interventions.^46^ SDG target 3.8 on achieving universal health coverage (UHC) aims to ensure that all people have access to quality health services, while also protecting against exposure to financial hardship.^47^ Financed by domestic public sources, UHC is a key strategic priority for strengthening health systems, and for the equitable and effective provision of needed, available, affordable and gender-sensitive health and social care.

UHC is based on concepts of equity, as set out in SDGs 1 (no poverty), 4 (quality education), 5 (gender equality), 8 (decent work and economic growth) and 16 (peace, justice and strong institutions). However, recent analysis has shown that even a well-functioning health-care system that is making progress towards UHC is not automatically equitable and gender-balanced.^48^ Unless explicit attention is paid to gender and the other intersectional drivers of inequalities, UHC may fail to improve equity. UHC may even exacerbate gender inequity since some groups of the population have greater health needs, but lower financing capabilities than others. Monitoring UHC focuses on two discrete components of health-system performance: levels of effective coverage; and financial risk protection.^49^ Table 1 provides some examples of how gender interacts with these two components.

In terms of effective coverage, health systems can monitor health inequalities covered by SDG target 5.1 (end all forms of discrimination against women and girls everywhere). They can also monitor other dimensions of inequity relevant in national contexts, such as defined in SDG 10 (reduced inequalities) and SDG target 17.18 (by 2020, enhance capacity-building support to developing countries…to increase significantly the availability of high-quality, timely and reliable data disaggregated by income, gender, age, race, ethnicity, migratory status, disability, geographic location and other characteristics relevant in national contexts).^50^ To achieve UHC, the health-system response must include comprehensive analyses to document who is being left behind in service access and to find out why.

The structures and processes of oppression and discrimination that exist in society will also play out within health systems.^51^ Entrenched gender-based discrimination affects the global health workforce, most whom are women.^34^ Discrimination in different areas of the health-care services is evidenced by gender pay gaps, lack of formal employment, physical and sexual violence, and lack of representation in leadership and decision-making.^50^ Health-system strengthening needs to better address how gender, power and social status can shape who is chosen as a health worker.^19^ At the very least, the health system should adopt good human rights practice to do no harm.^33^ It should ensure that it does not replicate or amplify local, often highly gendered, power dynamics that exclude or discriminate against certain population groups, including women, ethnic minorities and others.

## Synergies and interactions

While there is an implicit logic that the SDGs interact with and depend on each other, there is little consideration of how this works to support more coherent and effective decision-making to better facilitate monitoring, evaluation and evidence-informed action. A recent detailed analysis of interactions across the SDGs did consider SDG 3 along with SDGs 2 (zero hunger), 7 (affordable and clean energy) and 14 (conserve and sustainably use the oceans, seas and marine resources for sustainable development).^52^ However, the analysis did not contain detailed analysis of the interactions, enabling or otherwise, with SDG 5.

Another analysis looked at six sectors, family planning; maternal, newborn and child health; nutrition; agriculture; water, sanitation and hygiene; and financial services for the poor, across 76 studies in low- and middle-income countries.^53^ The study showed that gender equality and indicators of women’s and girls’ empowerment were associated with improvements in a variety of health and development outcomes. Furthermore, these associations were cross-sectoral, suggesting that to fully realize the benefits of promoting gender equality and women’s empowerment, the development community must collaborate in coordinated and integrated ways across multiple sectors.

## Conclusion

Realizing the right to health and well-being of all people by acting on existing gender inequities and their complex determinants is challenging. Several factors hinder progress. There is a tendency for government departments and development partners to take ownership of particular goals, including SDG 3. Action is needed that is multidisciplinary (for example, going beyond medicine to include social sciences, statistics or political economy), multisectoral (involving different sectors of government, not just the health sector or health ministry) and multistakeholder (going beyond government to include, for example, civil society, private sector and academia). It can be argued that SDG 5’s concept of gender is narrow, referring mainly to women and focusing on limited roles: as mothers, as caregivers and as victims of violence. There is little incentive therefore for countries to adopt a more holistic, gender-equal and progressively universal approach.^54^ Outdated understandings of gender fail to explicitly acknowledge and address the underlying power and hierarchy relations between men and women that shape their health through a complex interplay of health determinants, behaviours and health-system responses. More attention is also needed to how masculinities as collective patterns of behaviour affect men’s as well as women’s health.

Although studies and official statistics may strive to report sex-disaggregated statistical differences in health (as required in SDG target 17.18) this is not equivalent to showing the impact of gender in driving those differences. We need additional, more nuanced, qualitative analyses of gender influences in their specific contexts, involving participants as individuals. For example, studies that focus on understanding how sex-disaggregated differences are shaped by social inequalities and power differentials rooted in gender norms. Such analysis can also help shape the transformation of gender as it promotes or hinders equity as a means to health.

This paper has sought to unpack the complex relationship between gender and health equity across three domains: social determinants, health behaviours and health-system responses. These domains in turn will impact on, and be influenced by, progress across all SDGs, and not just SDGs 3 and 5. We conclude that those seeking to achieve SDG 3 need to shift their thinking and action in several areas, as outlined in Box 1. We propose an action agenda to improve health equity outcomes while also advancing gender equality and women’s empowerment. Adopting this agenda will accelerate progress for all people, in all their diversities, to realize to their fullest potential, their right to health and well-being across their life course.

Box 1 **Actions to promote gender-transformative approaches in the sustainable development goals to improve health**

1. Move beyond equating gender with women. Global, national and local health policy needs to take account of how the roles, behaviours, activities, attributes and opportunities of males and females are based on different levels of power. This understanding of gender as a social and relational construct of power amplifies inequities in health for everyone and intersects with other drivers of inequities.^55^

2. Adopt a holistic approach to analysis and action on gender. This approach will intersect with three domains of health: social determinants; health-seeking behaviour; and service delivery and health-system responses, and hence across the 2030 agenda for sustainable development.^12^ Applying gender to one of these domains alone will fail to address inequities in health efficiently.^13^

3. Invest in more gender analysis of sex-disaggregated data, alongside other stratifiers of social and health inequity. Global health journals should encourage authors to include a gender analysis of sex-disaggregated data, including how the social construction of masculinities and femininities shape men’s and women’s health.^37^

4. Acknowledge and act on the gendered nature of the health workforce. Formulate gender-sensitive policies and health professional regulations through all levels of health governance to ensure gender parity, increased leadership roles for women and decent conditions of work for all.^28^

5. Break down the isolated policy structures between different government sectors and programme areas and build a broad multi-stakeholder coalition for gender in global health. Such a coalition will aim to transcend narrow disease-focused approaches and engage more with civil society and with policymakers beyond ministries of health.^54^^,^^56^

6. Support transparency and accountability mechanisms at the country level. This can be done through strengthening a gendered health focus in voluntary national reviews, United Nations development assistance frameworks, and national health sector plans and programmes, building on the approach developed by Global Health 50/50.^28^
